# Giant Distal Anastomotic Pseudoaneurysm 35 Years after Bentall Operation Mimicking an “Elephant Trunk”

**DOI:** 10.1055/s-0041-1732399

**Published:** 2021-12-08

**Authors:** Raffaele Scaffa, Mario Torre, Antonio Longobardi, David Ferrara, Maria G. Vassallo, Francesco Itri, Enrico Coscioni

**Affiliations:** 1 Division of Cardiac Surgery, Azienda Ospedaliera Universitaria San Giovanni di Dio e Ruggi d'Aragona, Salerno, Italy

**Keywords:** aortic root, Bentall procedure, complications, pseudoaneurysm

## Abstract

We present the case of a giant distal aortic pseudoaneurysm 35 years after a classic mechanical Bentall operation. Computed tomography and coronary angiography showed that this originated from the distal suture line. The proximal suture and coronary ostia appeared to be intact. At reoperation, we found a complete dehiscence of distal suture line: the graft was floating in the pseudoaneurysm, mimicking an “elephant trunk” procedure. This complication suggested a systematic and accurate follow-up of patients who underwent an original Bentall procedure.

## Introduction

Pseudoaneurysm (also termed “false aneurysm”) of the aorta has been described in cases involving trauma, penetrating injury, ulcerative atherosclerotic plaques, and infection. However, pseudoaneurysm of the ascending aorta is also a rare but dreadful complication occurring several months to years after an aortic surgery (including the Bentall procedure). The diagnosis is often made by computed tomographic angiography (CTA). Pseudoaneurysms of the ascending aorta after the original inclusion/wrap technique of the Bentall procedure represent a difficult surgical management problem and are associated with substantial morbidity and mortality.

## Case Presentation

**Fig. 1:**
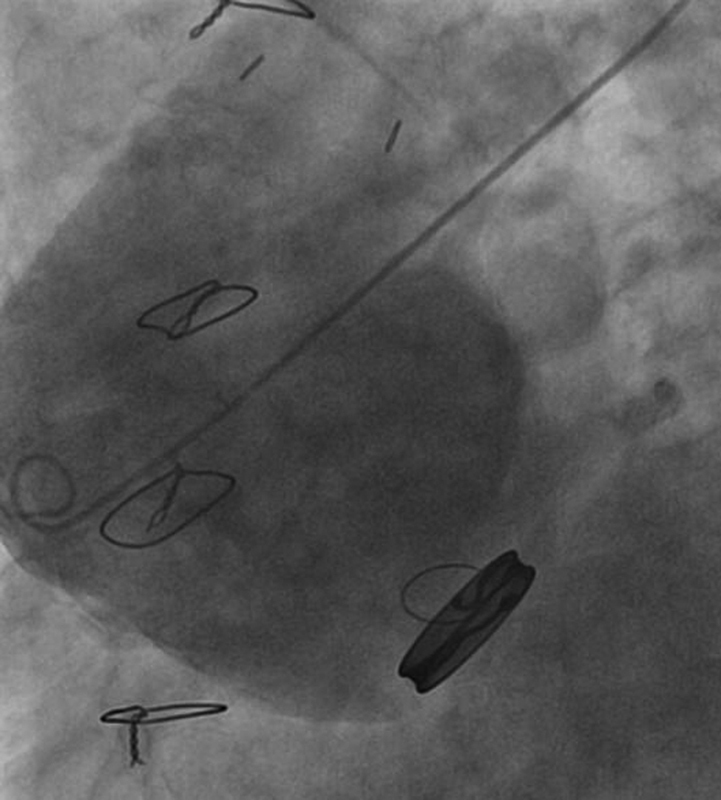
Aortography with the pig-tale catheter positioned directly inside the huge pseudoaneurysmatic sac, showing the origin from the distal Dacron graft anastomosis.

A 75-year-old man was referred to our Division after evidence of a giant pseudoaneurysm of the ascending aorta was visualized during coronary angiography (CA) performed to exclude an acute coronary syndrome ( Fig. 1 ). The patient's documentation clearly reported the classic Bentall-De Bono wrap/inclusion technique, 1 performed 35 years ago, with a composite graft consisting of a 27-mm Bjork–Shiley mechanical valve and a 34-mm tubular graft.

**Fig. 2:**
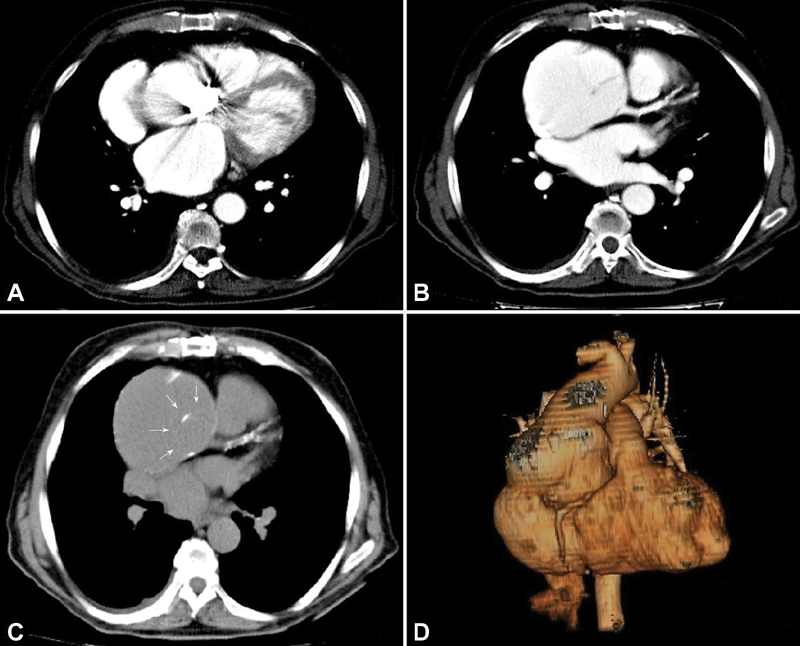
Computed tomography angiography scans revealed that both the coronary arteries ( **A, B** ) originated from the graft. The contour of the Dacron graft (white arrows) surrounded by pseudoaneurysm ( **C** ). Three-dimensional reconstruction ( **D** ).

At the current admission, the main finding on chest radiograph was an abnormal cardiothoracic ratio. The echocardiographic imaging confirmed a 9-cm diameter pseudoaneurysm of the distal ascending aorta, moderate intraprosthetic regurgitation, and an ejection fraction of 40%. Infections were excluded. At CA, the right main coronary artery was not imagined, and the left main could only be seen with hand injection into the proximal part of the conduit. CTA revealed that both the main coronary arteries were attached to the graft and displayed the contour of the Dacron graft surrounded by the massive distal pseudoaneurysm, as confirmed by three-dimensional reconstruction ( Fig. 2 ).

At reoperation, the right axillary artery and right femoral vein were cannulated to institute the cardiopulmonary bypass (CPB) and cool the patient down to 28°C. Resternotomy and adhesiolysis were performed. The aorta was cross-clamped in close proximity to the brachiocephalic trunk, and myocardial protection was achieved administrating Custodiol HTK (histidine–tryptophan–ketoglutarate) cardioplegia directly into the pseudoaneurysm. A vent was positioned in the left ventricle.

**Fig. 3:**
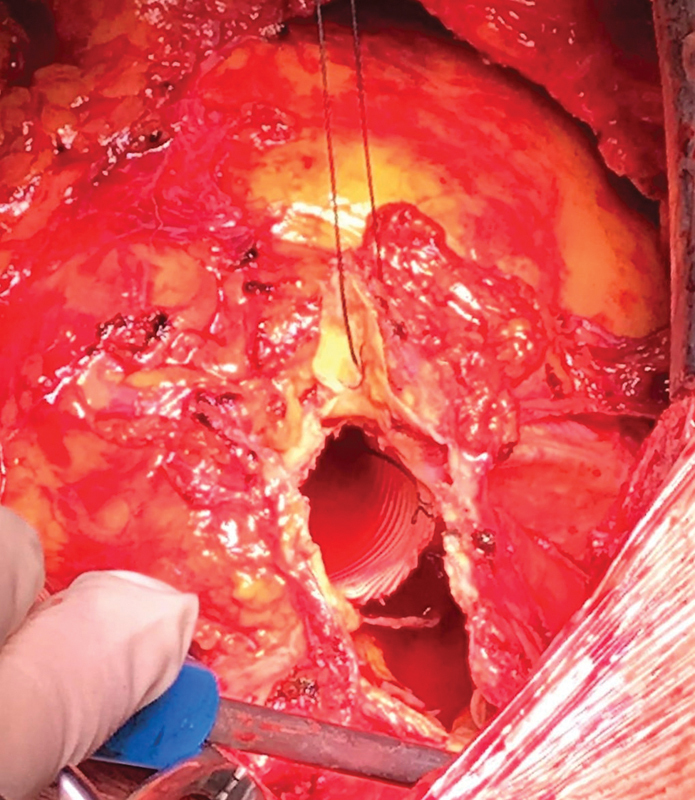
Intraoperative view: distal end of the graft floating in the pseudoaneurysm sac, mimicking an “elephant trunk.”

The pseudoaneurysm sac was then opened and excised. There was evidence of an almost total circumferential dehiscence of the Dacron graft along the distal suture line. The graft was floating in the pseudoaneurysm lumen mimicking an “elephant trunk” technique ( Fig. 3 ), whereby excess tubular graft material is left in the distal aorta during aortic arch repair. The prosthetic valve was still secured to the aortic annulus, but pannus formation was apparently interfering with the normal closure mechanism. Once explored, coronary ostia reached the graft and their suture lines were still intact, although sewn to frail tissue. Therefore, left and right coronary ostia were primarily mobilized, according to the button technique, 2 including a Dacron rim of the previously implanted graft.

After removing the mechanical valve, a biological composite valve conduit consisting of an Edwards Perimount Magna Ease 23 mm bioprosthetic valve and a GelweaweTM Valsalva 28-mm Dacron graft (Terumo Aortic, Renfrewshire, United Kingdom) was implanted. Interrupted 2/0 coated-braided pledgeted polyester sutures were placed through the aortic annulus. At the base of the graft, the collar was trimmed leaving a 5-mm remnant which was attached to the biological prosthesis, so that it was secured to the patient's annulus in a single-step fashion. Coronary ostia were resutured into the Valsalva graft skirt with 6/0 polypropylene. Finally, a distal anastomosis was performed with polypropylene 4/0 continuous suture in a normal portion of distal ascending aorta.

After rewarming, the patient was easily weaned from CPB. Postoperatively the patient was extubated 12 hours after surgery and recovered well.

## Discussion

Since it was introduced in 1968, the Bentall-De Bono technique 1 has continued to be considered the “gold standard” for treating combined aortic valve and aortic root pathology. Complications after aortic root surgery can be classified into thromboembolic or infective events (mainly secondary to the associated prosthetic valve replacement) and surgical complications related to the aortic conduit (bleeding, pseudoaneurysm, and anastomotic dehiscence). 3 According to a time-related evaluation, bleeding, and coronary artery kinking or torsion are usually early complications, while pseudoaneurysm, thromboembolic and infective events, and anastomotic dehiscence are usually observed at later follow-up. However, rates of aortic root reoperation after the Bentall procedure have decreased over the years.

Prevalence and natural history of pseudoaneurysm after the Bentall operation have not been stated. Their rare formation is typically described at the coronary ostial sites, 4 especially before the introduction of the button technique, 2 but they may form at any of the anastomotic sites. Few case reports describe a complete detachment of the proximal anastomosis, generally representing a perivalvular extension of endocarditis. Distal anastomotic pseudoaneurysm is even less commonly reported. 5 6

In our case, we ruled out an infectious origin and erosion of the aortic prosthesis or any primary lesions in the proximal coronary arteries. The continuous shear stress in the stiff and calcified ascending aortic graft could be the foremost reason for distal suture-line disruption and pseudoaneurysm formation. The development of pseudoaneurysm following ascending and/or root repair can be related to operative strategy and anastomotic technique. Specifically, risk factors associated with pseudoaneurysm after an original Bentall operation include suture-line tension, persistent bleeding into the perigraft space, and graft infection. Apparently, in our case, the inclusion/wrap technique might have prevented an acute rupture, although the pressure exerted by the blood collected between the aortic wall and the graft would lead to greater tension on the suture line. Kouchoukos et al 7 reported an increased occurrence of pseudoaneurysm formation after the inclusion/wrap technique. They first advocated abandoning the aortic wrap in favor of the open technique. In redo cases, the Valsalva graft can be a good option to facilitate coronary ostia reimplantation. This expedient (with the artificial Valsalva sinuses) might prevent coronary osteal pseudoaneurysms in all root surgery procedures by decreasing button suture-line tension. 8

The exact time of pseudoaneurysm formation in our case was unclear; the patient did not follow the recommendations for periodic echocardiographic follow-up. Appropriate imaging follow-up should never be neglected and very late life-threating complications should be anticipated. Because of the frail aortic condition, only noninvasive diagnostic techniques should be pursued; CTA and echocardiography correlate well, integrating their respective information.
